# Radioprotection of targeted and bystander cells by methylproamine

**DOI:** 10.1007/s00066-014-0751-9

**Published:** 2014-09-23

**Authors:** Susanne Burdak-Rothkamm, Andrea Smith, Pavel Lobachevsky, Roger Martin, Kevin M. Prise

**Affiliations:** 1 Centre for Cancer Research & Cell Biology, Queen’s University Belfast, Belfast, UK; 2 Molecular Radiation Biology Laboratory, Peter MacCallum Cancer Centre, VIC, Melbourne, Australia; 3 The Sir Peter MacCallum Department of Oncology, University of Melbourne, VIC, Melbourne, Australia; 4 Cellular Pathology, Oxford University Hospitals, Headley Way, Headington, OX3 9DU Oxford, UK

**Keywords:** Radioprotection, Methylproamine, Radiation-induced bystander effect, γH2AX, Radiotherapy, Radioprotektion, Methylproamine, Strahleninduzierter Bystander-Effekt, γH2AX, Strahlentherapie

## Abstract

**Introduction:**

Radioprotective agents are of interest for application in radiotherapy for cancer and in public health medicine in the context of accidental radiation exposure. Methylproamine is the lead compound of a class of radioprotectors which act as DNA binding anti-oxidants, enabling the repair of transient radiation-induced oxidative DNA lesions. This study tested methylproamine for the radioprotection of both directly targeted and bystander cells.

**Methods:**

T98G glioma cells were treated with 15 μM methylproamine and exposed to ^137^Cs γ-ray/X-ray irradiation and He^2+^ microbeam irradiation. Radioprotection of directly targeted cells and bystander cells was measured by clonogenic survival or γH2AX assay.

**Results:**

Radioprotection of directly targeted T98G cells by methylproamine was observed for ^137^Cs γ-rays and X-rays but not for He^2+^ charged particle irradiation. The effect of methylproamine on the bystander cell population was tested for both X-ray irradiation and He^2+^ ion microbeam irradiation. The X-ray bystander experiments were carried out by medium transfer from irradiated to non-irradiated cultures and three experimental designs were tested. Radioprotection was only observed when recipient cells were pretreated with the drug prior to exposure to the conditioned medium. In microbeam bystander experiments targeted and nontargeted cells were co-cultured with continuous methylproamine treatment during irradiation and postradiation incubation; radioprotection of bystander cells was observed.

**Discussion and conclusion:**

Methylproamine protected targeted cells from DNA damage caused by γ-ray or X-ray radiation but not He^2+^ ion radiation. Protection of bystander cells was independent of the type of radiation which the donor population received.

## Abstract (de)

**Einleitung:**

Radioprotektive Agenzien sind sowohl in der Strahlentherapie von Krebserkrankungen als auch im Strahlenschutz im Zusammenhang mit akzidenteller Exposition von Bedeutung. Methylproamine ist die Leitsubstanz einer Klasse von Radioprotektoren, die ihre Wirkung als DNA-bindende Antioxidanzien entfalten und so die Reparatur von transienten strahleninduzierten oxidativen DNA-Schäden ermöglichen. Die Studie untersucht die radioprotektive Wirkung von Methylproamine auf direkt bestrahlte und Bystander-Zellen.

**Methoden:**

T98G-Gliomzellen wurden mit 15 μM Methylproamine inkubiert und anschließend mit ^137^Cs-γ -Strahlen/Röntgenstrahlung bzw. He^2+^-Microbeam-Bestrahlung behandelt. Die Radioprotektion direkt bestrahlter und Bystander-Zellen wurde als klonales Zellüberleben oder im γH2AX-Foci-Assay gemessen.

**Ergebnisse:**

Eine radioprotektive Wirkung wurde für T98G-Gliomzellen nach direkter Bestrahlung mit ^137^Cs-γ-und Röntgenstrahlung beobachtet, nicht jedoch nach Bestrahlung mit He^2+^-Ionen. Der radioprotektive Effekt von Methylproamine auf die Bystander-Population wurde sowohl für Röntgenstrahlung als auch He^2+^-Microbeam-Bestrahlung untersucht. Die Bystander-Experimente für Röntgenstrahlen wurden mithilfe von Zellkulturmediumtransfers von bestrahlten zu unbestrahlten Zellkulturen durchgeführt, drei verschiedene Experimentdesigns wurden getestet. Radioprotektion wurde nur beobachtet, wenn die Empfängerkultur mit Methylproamine vorbehandelt wurden, bevor sie dem konditionierten Zellkulturmedium ausgesetzt wurden. In den Microbeam-Bystander-Experimenten wurden bestrahlte und unbestrahlte Zellen kokultiviert, und die Behandlung mit Methylproamine erstreckte sich kontinuierlich über den Zeitraum der Bestrahlung und die anschließende Inkubationszeit.

**Diskussion und Fazit:**

Methylproamine schützte direkt bestrahlte Zellen vor DNA-Schädigung durch γ-Strahlen und Röntgenstrahlung, jedoch nicht vor Schäden durch He^2+^-Ionen. Die Protektion der Bystander-Zellen war unabhängig von der Art der Bestrahlung, der die Donorpopulation ausgesetzt war.

## Introduction

**Fig. 1:**
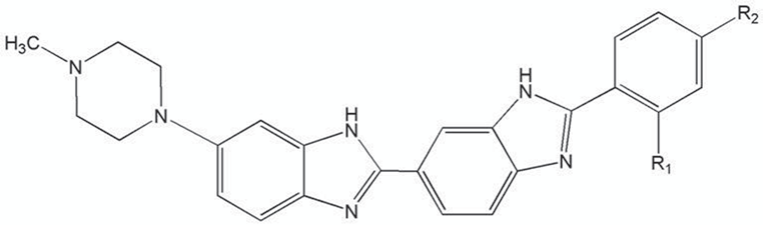
Methylproamine. The molecular structure of methylproamine and parent ligands are shown in Fig 1; for Hoechst 33342, R_1_ = H, R_2_ = OCH_2_CH_3_; for Hoechst 33258, R_1_ = H, R_2_ = OH; for methylproamine R_1_ = CH_3_ and R_2_ = N(CH_3_)_2_

Methylproamine is the lead compound of a new class of radioprotectors related to the commercially available fluorescent DNA stains Hoechst 33258 and Hoechst 33342 (see Fig. 1 for structures). Aside from potential uses in cancer radiotherapy, especially in the context of topical application to normal tissues “at risk” in cancer radiotherapy patients [1–3], such as oral mucosa, rectal mucosa, oesophageal mucosa and skin, there are potential applications for new radioprotectors that extend beyond the oncology arena, involving both planned and unplanned radiation exposures [4, 5].

The development of methylproamine and analogues was originally inspired by reports of unexpected radioprotective activity of Hoechst 33342 [6] and modification of the radiation sensitivity of human tumour cells by a bis-benzimidazole derivative was reported by Young et al. [7] and Denison et al. [8]. Guided by a mechanistic hypothesis for the radioprotective activity, analogues of Hoechst 33342 with more electron-rich substituents were synthesised and evaluated, and methylproamine proved to be a more potent radioprotector than Hoechst 33342 [9].

From a mechanistic standpoint methylproamine can be considered as a DNA binding anti-oxidant. Pulse radiolysis experiments support a mechanism involving repair of transient radiation-induced oxidative lesions on DNA, by a process of electron/hole transfer from/to nearby DNA-bound drug. This hypothesis is consistent with the results of recent investigations of double-stranded break induction, just an hour after irradiation, as reflected by the appearance of γH2AX foci, which serve as a sensitive marker for DNA double strand breaks [10]. As previously shown, treatment with methylproamine before and during irradiation reduced the level of radiation-induced γH2AX foci in cultured keratinocytes, in parallel with a subsequent reduction in the extent of radiation-induced cell-killing [11]. Thus the radioprotection by methylproamine, demonstrated for the clonogenic survival endpoint, can be ascribed to a decrease in the yield of early DNA damage as measured by the foci assay.

A major interest in contemporary radiobiology is the phenomenon of the bystander effect, which describes the observation that not only irradiated cells, but also the neighbours of the irradiated cells, respond to the effects of ionising radiation, particularly in terms of radiation-induced DNA damage, chromosome damage, mutation and loss of clonogenic viability [12–16]. The availability of microbeam technology has played a key role in the discovery and mechanistic investigation of the bystander effect. An alternative approach to investigate bystander effects are medium transfer experiments where filtered conditioned medium is transferred from irradiated cell cultures to nonirradiated (bystander) cultures. Proposed mechanisms for the propagation of bystander effects are the release of radical oxygen and nitrogen species, cytokines and the induction of a range of intra- and intercellular signalling pathways [17–23]. Radiation-induced bystander effects are currently discussed in the context of their contribution to carcinogenesis [24] and in view of a potential exploitation of bystander effects in cancer therapy [25]. The identification of molecular drug targets in the bystander signalling pathway provides a rationale for a pharmacological modulation of bystander effects [26].

We now report the results of microbeam and medium transfer experiments designed to explore radioprotection by methylproamine in the context of the bystander effect. In short, we address the question: Does methylproamine protect the targeted (irradiated) cells, the bystander cells, or both? Aside from extending the knowledge of the bystander phenomenon, the results also contribute to our understanding of the mechanism of radioprotection. T98G glioma cells were chosen for the study since the techniques to be used for the bystander studies were well-established for these cells. However it was then necessary to undertake separate experiments on the effect of methylproamine on these cells to determine the optimal drug concentration for the bystander studies. From our experience with V79 cells [6] and human keratinocytes [8], exposure to methylproamine is associated with concentration-dependent, and exposure time-dependent cytotoxicity. For example for a human keratinocyte cell line, a 1 h exposure to 20 μM methylproamine reduced clonogenic survival down to about 80 % [8]. Thus a suitable drug concentration was established for T98G cells (namely 15 μM), that provided radioprotection without intolerable cytotoxicity.

## Material and methods

### Experimental design

Radioprotection by methylproamine of directly irradiated and bystander cells was studied using γH2AX foci as the experimental endpoint for radiation-induced DNA damage. T98G cells were irradiated with He^2+^ ions or 240 kV X-rays. The radiation-induced bystander effect was studied in two different protocols—microbeam irradiation and medium transfer. The microbeam experiments used 3 MeV He^2+^ ions from the Gray Cancer Institute charged particle microbeam [27]. In the medium transfer experiments, donor cells were irradiated with 240 kV X-rays, and medium was transferred to recipient cells after contact with donor cells 30 min postirradiation. Recipient cells were fixed and processed for γH2AX 30 min later.

Protection of bystander cells was investigated in medium transfer experiments, using three different designs with respect to the addition of methylproamine (15 μM):

- addition to the donor cell population before irradiation and transfer with the filtered conditioned medium,
- addition to the filtered conditioned medium before transfer to the recipient cells or
- pre-incubation with the recipient cell population as well as addition to the transferred medium.

Methylproamine was added to T98G cells to a concentration of 15 μM at 15 min prior to irradiation (with exception of the medium transfer experiments where the specific treatment protocol is detailed above) and was not removed until 30 min after irradiation when the cells were fixed and processed for γH2AX immunohistochemistry.

In separate experiments it was established that clonogenic survival for T98G cells exposed to 15 μM methylproamine was > 50 % (data not shown) and the protection of T98G cells from ^137^Cs γ-ray irradiation by treatment with 15 μM methylproamine was shown in a clonogenic assay.

### Cell culture conditions

T98G glioma cells were cultured in RPMI 1640 medium (Cambrex, Verviers, Belgium) supplemented with 10 % FBS (PAA, Pasching, Austria), 2 mM L-glutamine, 100 units/ml penicillin and 100 µg/ml streptomycin (all Cambrex, Verviers, Belgium). T98G cells were obtained from the European Collection of Cell Cultures (ECACC). Cells were incubated at 37 ℃, 5 % CO_2_ and for all experiments nonconfluent cell cultures were used.

### Microbeam irradiation

The Gray Cancer Institute charged particle microbeam system was used for targeted irradiation [27, 28]. Cells were seeded in the centre of Mylar foil dishes pretreated with 1.7 µg/cm^2^ BD Cell-Tak adhesive (Becton Dickinson, Erembodegem, Belgium) and stained in medium containing a 0.8 µM concentration of the fluorescent dye Hoechst 33342 (Molecular Probes, Leiden, the Netherlands) to enable visualisation of cell nuclei by fluorescent microscopy at the microbeam stage.

For the study of targeted cells, 1,000 cells were seeded within a volume of 30 µl of complete culture medium in the central area of the dish and irradiated with a defined number of He^2+^ ions per nucleus.

Bystander cells were studied as cells adjacent to cells irradiated by a line of 5 helium ions/µm (approx. 2–3 μm wide) across the culture dish; for bystander experiments 10,000 cells were seeded in the centre of each dish.

Cells were incubated at 37 ℃, 5 % CO_2_ for 30 min after irradiation and prior to fixation.

### X-ray irradiation and medium transfer experiments

Cells were irradiated with 240 kV X-rays (Pantak) with 4.3 mm aluminium filtration (0.4 Gy/min). Cells were grown on coverslips sitting in tissue culture dishes. Irradiation and subsequent incubation was performed at 37 ℃ to allow foci formation. After 10 min, cells were placed on ice to prevent DNA repair, fixed with 4 % paraformaldehyde and immunostained for γH2AX foci as described below.

For medium transfer experiments, cells were seeded on 22 × 22 mm^2^ coverslips placed in 6-well tissue culture dishes and were treated with filtered medium obtained from cells, which had been irradiated with 2 Gy of X-rays (Pantak, 240 KV) followed by 30 min of incubation. The cell culture medium was filtered through a 0.45 μm syringe filter in order to prevent the transfer of cells with the supernatant culture medium. The recipient cells were incubated with the conditioned medium at 37 ℃, 5 % CO_2_ for further 30 min.

### Immunocytochemistry

For immunocytochemistry, cells were fixed for 15 min with 4 % paraformaldehyde, permeabilized with 0.5 % Triton-X 100 (both Sigma, Poole, UK) and blocked with 3 % FBS (PAA, Pasching, Austria) in PBS for 30 min at room temperature. Incubation with a primary antibody specific for γH2AX (H2AX p139S; Upstate, Chandlers Ford, UK) for 1 h at room temperature was followed by incubation with a matching Alexa Fluor 488 labelled secondary antibody (Molecular Probes, Leiden, the Netherlands) and nuclear counterstaining with DAPI. Between antibody incubations, cells were washed tree times with PBS/3 % FBS. For cells cultured on coverslips, the inverted coverslip was placed on a glass slide with VectaShield mounting medium for fluorescence microscopy (Vector Laboratories, Burlingame, CA) after staining was completed, and the edges were sealed with clear nail varnish. Cells growing on Mylar foil were stained within the microbeam dish and the foil was glued to a glass slide. A coverslip was placed on top of the Mylar foil with VectaShield mounting medium after staining was completed, and the edges were sealed with nail varnish.

A fluorescence microscope was used for imaging and analysis (Zeiss, Welwyn Garden City, UK). For quantification of foci numbers, individual γH2AX foci per cell nucleus were counted by eye.

### Clonogenic survival assay

Cultures for use in the clonogenic survival assays were in log phase growth with approximately 1 × 10^6^ cells/25 cm^2^ flask. The cell number to media volume ratio was kept constant for all experiments (1 × 10^6^ cells/5 ml media). Accordingly, the media volume in the experimental flasks was adjusted after an initial cell number estimation using duplicate spare flasks. Methylproamine was solubilised in 40 mM acetic acid/50 % ethanol and added to the medium in the flasks to give a final concentration of 15 μM. The flasks were then incubated for 30 min at 37 ℃, 5 % CO_2_ before being irradiated with ^137^Cs γ-rays using a Gamma Cell 40 Irradiator (Nordion International, Ottawa, ON, Canada) at a dose rate of 0.56 Gy/min. Control flasks (containing no methylproamine) were exposed to doses between 0 and 12 Gy while the flasks containing methylproamine were exposed to doses of 0–24 Gy. Following irradiation, flasks were returned to 37 ℃, 5 % CO_2_ for the remainder of the incubation period (1 h total incubation).

After the incubation, cells were washed with PBS/EDTA and the monolayer disrupted by treatment with 0.01 % pronase (Merck KGaA, Darmstadt, Germany) in PBS/0.5 mM EDTA (Sigma, St Louis, MO, USA). Following pronase treatment, the pronase was neutralised by the addition of growth media and a single cell suspension was created by repeat pipetting (20 ×) with a 5 ml pipette. The cell suspension was then centrifuged at 1500 rpm (458 rcf) for 5 min, the cell pellet resuspended in growth media and again repeatedly pipetted (20 ×) to form a single cell suspension. Cell numbers were determined using a Z2 Coulter Counter (Beckman Coulter Australia, Gladesville, NSW, Australia).

After determining the cell numbers in each flask, cells were diluted, using serial dilutions, to the appropriate concentration and plated into 60 mm petri dishes. Five replicates were made from each flask. The petri dishes were then incubated, undisturbed, for 8 days at 37 ℃ in a humidified atmosphere of 5 % CO_2_. The resulting colonies were fixed with neutral formalin before being stained with 0.01 % crystal violet. Colonies containing more than 50 cells were counted by hand using an inverted stereo microscope.

## Results

### Effect of treatment with methylproamine on directly irradiated cells

**Fig. 2:**
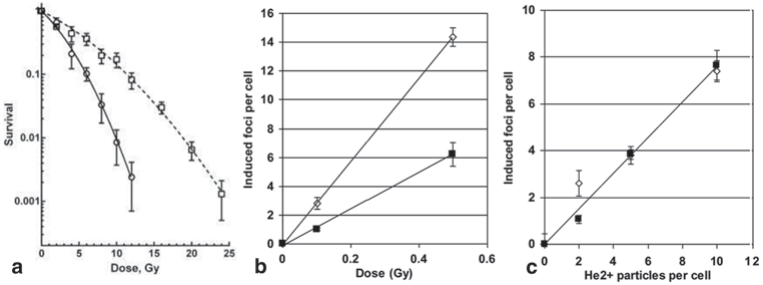
**a** Clonogenic survival curves for T98G cells after ^137^Cs γ-ray irradiation, with (*open squares*) and without (*open circles*) methylproamine treatment (15 µM; 15 min prior to irradiation). The data points show the average and standard deviation for three separate pairs of survival curves. The dose-modifying factor is 2.1. **b** Effect of methylproamine on γH2AX foci induction by X-ray irradiation. 15 µM methylproamine added 15 min before radiation (X-ray, 240 kV). Methylproamine significantly protected against radiation-induced foci in “targeted” T98G cells (*p* < 0.01, t-test), in keeping with the results shown for the clonogenic survival endpoint. A total of 1006 cells were evaluated. The background foci level in the control cell population was subtracted from the foci count. Error bars represent the standard error of the mean (*SEM*). **c** Methylproamine treatment (15 µM, added 15 min before radiation) had no effect on γH2AX foci induction by He^2+^ microbeam irradiation in “targeted” T98G cells. A total of 336 cells were evaluated. The background foci level in the control cell population was subtracted from the foci count. Error bars represent the SEM

Clonogenic cell survival was determined for T98G glioma cells after single doses of ^137^Cs γ-ray irradiation between 0 and 12 Gy for cells not treated with methylproamine and doses between 0 and 24 Gy for those protected by methylproamine treatment. Treatment with 15 μM of methylproamine—prior to, during and after irradiation—significantly increased the clonogenic survival of T98G cells. On the basis of these experiments, the extent of radioprotection corresponded to a dose modification factor of 2.1 (Fig. 2a), similar to the results published for the FEP1811 human keratinocyte cell line [9, 11].

In order to visualise and quantify radiation-induced DNA damage and the potential protection by methylproamine treatment, the γH2AX assay was applied which has been previously used as suitable endpoint in bystander studies [15, 29, 30]. T98G glioma cells were irradiated with X-ray doses between 0 and 0.5 Gy and immunofluorescent staining for γH2AX foci was performed (Fig. 2b). Treatment with 15 μM of methylproamine—continuously prior, during and after irradiation—significantly reduced the induction of γH2AX foci per cell (*p* < 0.01, t-test).

In order to study the effect of methylproamine on DNA damage induced by heavy charged particle irradiation on directly targeted cells, cultures of T98G glioma cells were irradiated with 0–10 particles of He^2+^ ions per nucleus using the Gray Cancer Institute charged particle microbeam system. A linear increase in the number of γH2AX foci per cell with increasing radiation dose was observed (Fig. 2c). Treatment with methylproamine could not reduce the number of He^2+^-induced γH2AX foci in directly irradiated cells, indicating that no protection was achieved from DNA damage by He^2+^ ion irradiation.

### Protection of bystander cells from radiation-induced DNA damage by methylproamine

To investigate the effect of methylproamine on X-ray-induced γH2AX foci formation in bystander cells, a medium transfer assay was applied and filtered cell culture medium was transferred from an irradiated cell culture to a nonirradiated one. Three different treatment protocols were chosen: Methylproamine was (1) added to the donor cell population before irradiation and transferred with the filtered conditioned medium, (2) added to the filtered conditioned medium before transfer to the recipient cells, or (3) pre-incubated with the recipient cell population and also added to the transferred medium.

**Fig. 3:**
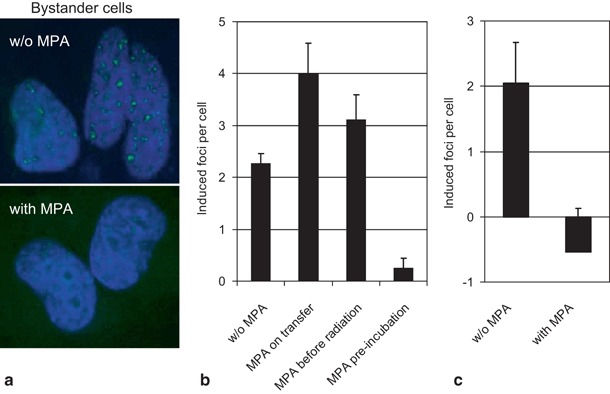
Effect of methylproamine (*MPA*) on the induction of γH2AX bystander foci by medium transfer from X-ray irradiated cells and He^2+^ microbeam bystander cultures. **a** Bystander γH2AX foci are present in T98G cells treated with conditioned medium derived from X-ray (2 Gy) irradiated cells. Pretreatment with methylproamine markedly reduced γH2AX bystander foci induction. **b** A significant X-ray (2 Gy) induced bystander effect was seen in cells without methylproamine treatment (column 1; *p* < 0.01, t-test). Three different protocols were applied for the investigation of the effect of methylproamine on the bystander effect: Methylproamine added to the filtered conditioned medium before transfer to the recipient cells (column 2), added to the donor cell population before irradiation and transferred with the filtered conditioned medium (column 3), or pre-incubated with the recipient cell population and also added to the transferred medium (column 4). Pretreatment of recipient cells with methylproamine (15 µM) suppressed foci induction by conditioned medium from X-ray irradiated cells (*p* < 0.05, t-test) whereas the two alternative treatment protocols without pre-incubation had no significant effect. A total of 1474 cells were evaluated. The background foci level in the control cell population was subtracted from the foci count. Error bars represent the standard error of the mean (*SEM*). **c** Methylamine treatment (15 µM) prevented He^2+^ microbeam irradiation-induced γH2AX bystander foci. A total of 414 cells were evaluated. The background foci level in the control cell population was subtracted from the foci count; the cause of the small negative value for the “induced foci” in the methylamine-treated sample is the correction for background levels. Error bars represent the SEM

T98G cells that were incubated with filtered medium derived from the irradiated donor cells showed significant induction of bystander γH2AX foci [Fig 3a (upper image) and 3b (column 1)], consistent with our previous studies [14, 15]. Following treatment with methylproamine according to the three different protocols detailed above, a significant reduction of medium transfer-induced bystander γH2AX foci was only seen when the recipient bystander cells were pre-incubated with 15 μM of methylproamine and methylproamine was also added to the transferred filtered medium (*p* < 0.01, t-test) [Fig. 3a (lower image) and 3b (column 4)]. The two other protocols where the donor cells were pretreated or the agent was only added at the time of medium transfer did not prevent the induction of γH2AX foci in the recipient bystander cell population (Fig. 3b, columns 2 and 3). This observation supports the hypothesis that methylproamine has to form a complex with the DNA molecule in order to protect the DNA from oxidative damage.

In order to study the protection of bystander cells from radiation induced DNA damage by heavy charged particle irradiation, T98G cells were irradiated with a line of 5 He^2+^ ions per μm, hitting cells growing in the area of this target line. Bystander cells, i.e. cells adjacent to this line of irradiated cells, showed a marked increase in the average numbers of γH2AX foci per cell (Fig. 3c; column 1). Treatment with 15 μM methylproamine lead to a significant reduction of He^2+^ -induced γH2AX foci in the bystander cells (column 2; *p* < 0.05, t-test), similar to the effect of methylproamine observed for the bystander population in X-ray treated cells.

## Discussion

The results presented in this study confirm that methylproamine protects cultured cells from radiation-induced DNA damage. While the protective effect for directly irradiated cells was dependent on the quality of the irradiation and applied to X-irradiation but not to irradiation with He^2+^ ions, bystander cells were shielded from indirectly induced DNA damage in both settings.

The cytotoxic effect of ionizing radiation is elicited largely by DNA damage attributed to the production of highly reactive radicals such as hydroxyl and superoxide radicals, in combination with direct ionisation events in DNA. Different qualities of ionising radiation cause different patterns of DNA damage. Low linear energy transfer (LET) radiation such as X-rays and γ-rays causes dispersed DNA damage including single strand breaks, base oxidation and occasional double strand breaks. In contrast, irradiation with high LET charged particles results in clustered DNA damage with frequent double strand breaks and complex patterns of DNA damage [31].

In order to understand the mechanisms underlying the radioprotective effect of methylproamine in directly targeted and bystander cell populations, these results need to be discussed in the context of the hypothesised mode of action of methylproamine. Based on pulse radiolysis studies it is concluded that radioprotection by DNA-binding ligands like methylproamine is mediated by repair of a transient oxidising lesion on DNA, by electron/hole transfer [32]. It is proposed that after direct or indirect ionisation of a base, or base pair [33], the “injected” hole migrates along DNA [34]. The bound ligand acts as a reducing agent, contributing an electron to neutralise the hole. In the context of DNA damage and cytotoxicity, the initial lesion is evidently more damaging than the oxidised ligand species. The requirement for pre-incubation of cells with methylproamine prior to exposure to X-ray irradiation or conditioned medium supports this hypothesis as complex formation between radioprotective agent and DNA molecule is required. Oxidative DNA damage is an important mechanism for the cytotoxicity of ionising radiation and also for bystander effects. In bystander cells it is postulated that increased levels of oxidised DNA damage leads to an increased probability of stalled replication forks and consequential DNA DSB [15]. Methylproamine can quickly repair oxidised lesions if it is closely associated with the DNA molecule at the time of radiation. However, with high LET radiation, the molecular density of clustered lesions could exceed the capacity of the charge transfer mechanism. Repair of each lesion requires a separate DNA-bound methylproamine molecule, and a limited number of bound ligand molecules will be within the physical range of the charge transfer process. This expectation of a limited ability of methylproamine to confer radiation protection of high LET radiation provides an explanation for an early report [35] of poor radioprotection by proamine [a close analogue of methylproamine; Fig 1; R_1_ = CH_3_ and R_2_ = N(CH_3_)_2_] of cultured cells irradiated by thermal neutrons in the presence of ^10^B-borate (boron neutron capture generates high LET fission products) in the cell culture medium.

Based on the results of the present study, it is concluded that methylproamine protects both directly targeted and bystander cell populations from DNA damage induced by X-ray irradiation. However for high LET radiation, bystander but not targeted cells are protected by methylproamine. The chemical identity of the transient lesions involved in protection of targeted cells is the subject of speculation. Much less is known about the chemical nature of DNA lesions induced in bystander cells, compared to the considerable data for targeted cells. Accordingly, even less can be proposed about the mechanism of protection of bystander cells by methylproamine.

Interestingly, the treatment with methylproamine reduced the number of background foci in unirradiated T98G glioma cells (data not shown) suggesting that intrinsic ROS levels contribute to background level of γH2AX foci in a given tumour cell line.

Protective effects of methylproamine and related agents may also have relevance in other situations where oxidative stress mediated responses are involved. ROS-mediated genotoxic effects are thought to play a central role in processes like aging and carcinogenesis. Recently, ROS/NOS- and cytokine-mediated DNA damage detected by the γH2AX assay was described in chronic inflammatory conditions, aging/senescence and in association with cancer [36–40].

There has been significant interest in the potential role of bystander signalling in therapeutic response both with a view to determining its clinical impact on tumour cell killing and potential carcinogenic effects in surrounding normal tissues [25, 26]. If these responses are potentially deleterious, agents such as methyproamine could play a role in treatment strategies for the use of ion beam approaches to preferentially protect surrounding normal tissues from the impacts of bystander signalling.

It is an interesting thought that radioprotective agents like methylproamine may also become a useful therapeutic option to prevent DNA damage caused by genotoxic stress in contexts other than radiation exposure.
